# Entirely enzymatic nanofabrication of DNA–protein conjugates

**DOI:** 10.1093/nar/gkx707

**Published:** 2017-08-16

**Authors:** Giulio Bernardinelli, Björn Högberg

**Affiliations:** Department of Medical Biochemistry and Biophysics, Karolinska Institutet, 17 177 Stockholm, Sweden

## Abstract

While proteins are highly biochemically competent, DNA offers the ability to program, both reactions and the assembly of nanostructures, with a control that is unprecedented by any other molecule. Their joining: DNA–protein conjugates - offer the ability to combine the programmability of DNA with the competence of proteins to form novel tools enabling exquisite molecular control and the highest biological activity in one structure. However, in order for tools like these to become viable for biological applications, their production must be scalable, and an entirely enzymatic process is one way to achieve this. Here, we present a step in this direction: enzymatic production of DNA–protein conjugates using a new self-labeling tag derived from a truncated VirD2 protein of *Agrobacterium tumefaciens*. Using our previously reported MOSIC method for enzymatic ssDNA oligo production, we outline a pipeline for protein–DNA conjugates without the need for any synthetic chemistry in a one-pot reaction. Further, we validate HER2 staining using a completely enzymatically produced probe, enable the decoration of cell membranes and control of genetic expression. Establishing a method where protein–DNA conjugates can be made entirely using biological or enzymatic processing, opens a path to harvest these structures directly from bacteria and ultimately *in-vivo* assembly.

## INTRODUCTION

Achieving a successful DNA–protein conjugate, despite the availability of several different approaches (1–7), remains a pitfall-prone process that is highly protein dependent and requires optimizations for each new conjugate. Over-conjugation, target protein loss of function or low reaction yield are some of the most common problems. To avoid such issues, conjugation strategies based on genetically encoded self-labeling tags, have been recently developed to enable a reproducible site specific labeling of the target proteins (8,9). Nevertheless, for any of these common strategies, the DNA molecule of interest needs to be chemically modified as well—a process that is only feasible when starting out with synthetic DNA oligonucleotides.

Among the plethora of naturally occurring proteins some have evolved a very peculiar function: the ability to establish a covalent bond at a specific sequence location of an unmodified nucleic acid molecule with the use of divalent ions as sole cofactor (10). Such proteins are involved in genetic material transfer from a pathogen to its host (11), in DNA digestion (12), remodelling (13,14) and replication (15). The capability of these proteins to form strong bonds is essential to avoid a loss of their DNA cargo while subjected to mechanical stresses.

One such protein is VirD2 from the plant parasite *A. tumefaciens* (16). This enzyme has been extensively characterized due to its crucial importance in the pathogenic process in which it acts as pilot protein, guiding the infectious DNA to the nucleus of the host. The VirD2 domain involved in the covalent bond formation is located within the first 200 amino acids of the protein and its core is made of a tyrosine residue (Y29) that bridges the protein to the DNA (17). The discovery and characterization of this mechanism represented a milestone for plant molecular biology. In fact, this microorganism DNA transfer machinery enabled a facile generation of recombinant plant organisms (18). The small size and efficacy of VirD2 and other similar relaxase enzymes, carrying out the conjugation to DNA molecules, have been exploited for other biotechnological purposes as, for example, to generate conjugates applied in DNA nanotechnology (19), to link DNA–protein information (20) and as biosensors (21).

**Figure 1.**
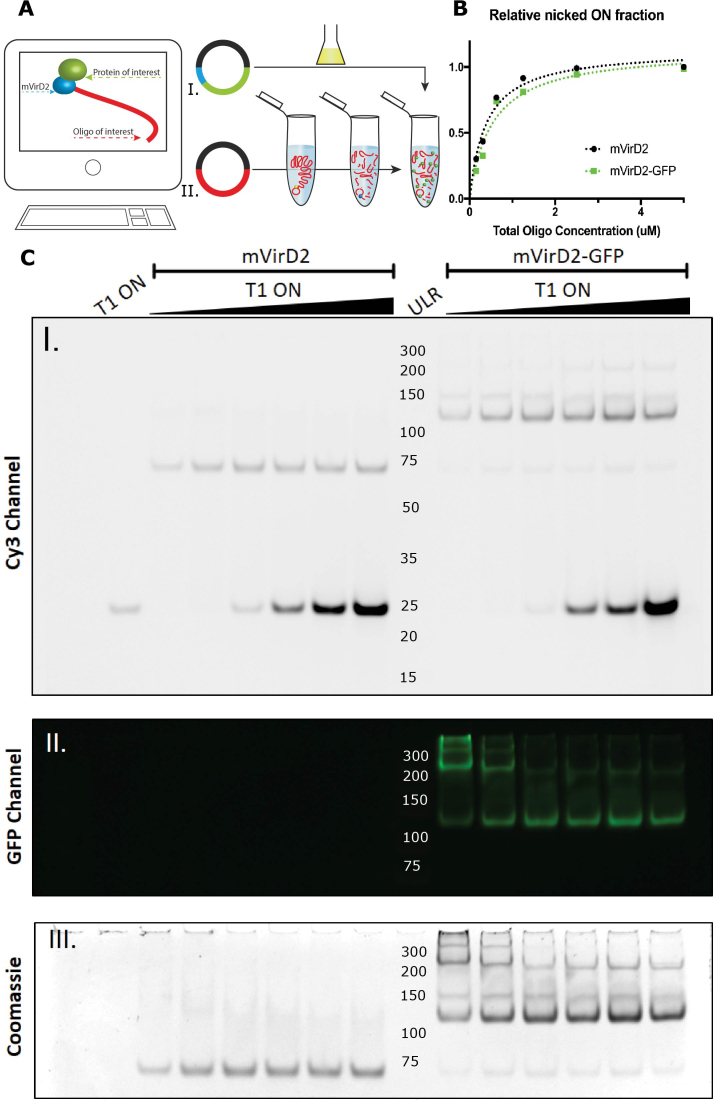
mVirD2 as a new self-tagging domain for protein-ssDNA conjugation. Schematic representation of the conjugation protocol (**A**) where the wanted conjugate is first designed *in-silico*. The protein of interest (POI) fused with the mVirD2 self-tagging domain is overexpressed and purified (**A I**.). In addition (**A II**.) the plasmid encoding the ON of interest (OOI) is processed following the MOSIC protocol (pseudogene re-circularization, Rolling Circle Amplification, digestion). Finally, the POI fusion protein is added to the MOSIC product to obtain the conjugate. As a proof of concept, the purified minimal relaxases domain (mVirD2) and the fusion product (mVirD2-GFP) are compared aside in a 10% native polyacrylamide gel (**C**) using a 3′ Cy3-labeled synthetic ON (T1) containing the consensus sequence for the conjugation reaction. ULR: ultralow range ladder (Thermo Fisher). A fixed equivalent amount of protein was incubated with increasing concentration of T1 ON for 1 h at 37°C. The same gel is first imaged for Cy3 (**C I**.) where it is possible to observe the formation of a DNA–protein complex. The same gel is then imaged for the GFP (**C II**.) where the increase of oligonucleotide correspond to a higher degree of complex association that influences the protein migration. The gel is also stained for total nucleic acids (Supplementary Figure S5) before being stained with colloidal Coomassie for total proteins. According to the nicked fragment generated in the conjugation reaction the activity of the two purified protein is compared (**B**).

Herein, we propose to genetically fuse any protein of interest to a newly cloned minimal domain of the bacterial relaxases VirD2 as a self-tagging domain. This will enable fast, and site specific, conjugation of *unmodified* DNA molecules in physiological conditions. After initial characterization using synthetically produced ssDNA (single-stranded DNA), we have been able to apply this conjugation procedure in the method previously developed by our lab for the enzymatic production of high quality oligonucleotides (ONs) of any length (MOSIC) (22,23) without any buffer exchange in a one-pot reaction (Figure 1A). We argue that this type of completely enzymatic production of DNA–protein conjugates is an important step towards a large-scale production, of such entirely biologically assembled devices and would be of use for many of the emerging applications in bionanotechnology (24–26), molecular logic (27) and detection applications (28,29) where DNA attached to proteins form a crucial component.

## MATERIALS AND METHODS

### Plasmid construction

The sequence encoding the minimal VirD2 protein was amplified by PCR from pSDM3149 (a generous gift from Prof. Van Der Zaal (17)) and cloned (EcoRI/SmaI) under the control of a T7 promoter into pSNAP-tag (T7)-2 (NEB) becoming pmVirD2. pmVirD2-GFP was obtained by overlap extension PCR cloning as previously described (30). Details about gene sequences and cloning procedures are described in Supplementary Information S1.

### Proteins expression and purification

Transformed BL21 (DE3) T1R p RARE2 were grown over night at 30°C, 175 RPM. The next day, the cultures were refreshed in 3 L TB and cultured at 37°C until OD 2. The temperature was subsequently set at 18°C and the cells induced at OD 3 with IPTG (final concentration 0.5 mM). Protein expression continued overnight before the cells were harvested by centrifugation (10 min at 4500 × g). Cell pellets were re-suspended in 4°C IMAC lysis buffer (100 mM HEPES, 500 mM NaCl, 10% glycerol, 10 mM imidazole, 0.5 mM TCEP, pH 8.0) with the addition of one tablet (pre-dissolved in 2 ml H_2_O) cOmplete, EDTA-free protease inhibitor cocktail (Roche) and 5 μl benzonase nuclease 250 U (Sigma-Aldrich) per culture. The resuspended cells were disrupted by pulsed sonication (4 s/10 s 3 min, 80% amplitude). The sonicated lysate was centrifuged (20 min at 49 000 × g), the supernatant was filtered through 0.45 μm filters and loaded onto the ÄKTA Xpress and purified overnight using a 5 ml HisTrap HP IMAC column (GE Healthcare) washed with 20 mM HEPES, 500 mM NaCl, 10% glycerol, 10 mM imidazole, 0.5 mM TCEP, pH 7.5 and eluted with 20 mM HEPES, 500 mM NaCl, 10% glycerol, 500 mM imidazole, 0.5 mM TCEP, pH 7.5. An additional gel filtration step using a HiLoad 16/60 Superdex 75 column (GE Healthcare) using TKM buffer (50 mM Tris–HCl pH 8, 150 mM KCl, 1 mM MgCl_2_, 10% glycerol).

### Oligonucleotides, pseudogene design and synthesis

The ONs were designed to minimize secondary structures using Nupack (31) (http://www.nupack.org) and purchased from Integrated DNA Technologies, Leuven Belgium. The Same *in-silico* evaluation was conducted for the MOSIC (Monoclonal Stoichimetric (22)) ONs. Selected sequences of interest were fused with the 5′ target sequence (T1) and inserted in MOSIC pseudogenes with the help of the online tool available on our website (http://www.hogberglab.net/software/).

### MOSIC oligonucleotides production

The plasmids enclosing the oligonucleotide pseudogenes (GeneArt, Thermo Fisher) were transformed by heat shock in subcloning efficient Dh5α (Invitrogen) and grown overnight in 10 ml LB media supplemented with 100 μg/ml ampicillin. The plasmids were purified the next day using the GeneElute HP plasmid miniprep kit (Sigma). 20 ug of the obtained plasmid were digested for 4 h with BsaI-HF (NEB) in 100 μl with the addition of 10 μl of CutSmart buffer (NEB) and the psudogenes extracted from a 0.7% agarose gel, 0.5× TAE, using illustra GFX PCR DNA and gel band purification kit (GE Healthcare). The pseudogenes were processed to produce single-stranded ONs as previously described (22). For the protein gel, the crude MOSIC product was cleaned from most of the proteins after the digestion using the QuiaEXII kit (Qiagen) according to the instructions of the manufacturer.

### Conjugation reaction

The conjugation reaction for electro-mobility shift assay gels with labeled ONs, were made in 10 μl volume reactions. Fixed amounts of mVirD2 or mVirD2-GFP protein (250 ng if not differently indicated) were incubated with the ON in TKM buffer for 1 h at 37°C in a PCR thermocycler. The reaction with MOSIC ONs was also carried out in 10 μl using the crude MOSIC mixture or the protein depleted (clean-up with the QuiaEXII Kit, Qiagen) mixture with addition of mVirD2 or mVirD2-GFP (500 ng).

### Gel analysis

All conjugations were analyzed on polyacrylamide gels. For native gels a fresh solution of polyacrylamide (19:1) final concentration 10% (Biorad), TBE buffer, final concentration 0.5× and glycerol (2.5%, v/v) was mixed and polymerized by the addition of fresh 10% APS solution, final concentration 10%, and TEMED, final concentration 1%, in a mini-protean gel system (Biorad). The samples were loaded in the wells directly after the conjugation reaction without the addition of further loading buffer, TBE 0.5× was used as running buffer and the gels were run at room temperature for 30 min at 40 V/cm. MOSIC ONs were quality assessed before the conjugation reaction by Urea-PAGE (8 M urea, polyacrylamide 29:1 final concentration 10% and TBE final concentration 1×) TBE 1× was used as running buffer and ran at room temperature for 40 min at 40 V/cm. Urea-PAGE gels were imaged right after electrophoresis for eventual intrinsically labeled ON then post-stained with SYBR gold (Invitrogen) 1:10 000 in TBE 1× buffer for 20 mins before imaging and finally with colloidal Coomassie reagent GelCode blue stain reagent (ThermoFischer). Protein electrophoresis was carried out using a bis-tris gel (stacking: 6% polyacrylamide, resolving: 12% polyacrylamide 27.5:1 (Biorad), 0.33 M Bis-tris buffer pH 6.5) using 100 mM MES, 100 mM Tris, 5 mM EDTA and 0.5% SDS as running buffer. Such gels were run at 26.6 V/cm for 1 h and stained with colloidal Coomassie or Sypro orange (Thermo Fisher).

### Enzymatic Her2 aptamer conjugate characterization

The enzymatically produced HeA2_3 aptamer-mVirD2-GFP conjugate was assessed on a denaturing PAGE as previously described. Moreover, the binding ability to its target was tested *in-vitro*. Different amount (from 500 to 15 ng) of the recombinant Fc-tagged HER2 protein (Thermo Fisher) were first immobilized on protein G decorated dynabeads (Thermo Fisher) and then incubated at room temperature for 15 min in PBS in triplicate with a fixed amount (160 ng) of mVirD2-GFP pre-conjugated with the aptamer. After extensive washes of the magnetic beads the relative GFP fluorescence was recorded on a Varioskan Lux plate reader (Thermo Fisher).

### Cell cultures and dot blot

SK-BR-3 cells (a gift from Ana Texeira's lab) were cultured in DMEM/GlutaMAX (Gibco) supplemented with 10% FBS (Sigma), 1% Pen/Strep (Sigma) and maintained at 37°C 5% CO_2_ atmosphere. For the staining experiment, 15 000 cells were seeded per well in a Millipore 8-well EZ slide. Twenty four hours later, the cells were washed with PBS, fixed with 4% PFA for 10 minutes at room temperature and washed three times every 5 min with PBS. Nuclei were stained with Hoechst 33342 (Thermo Fisher) at the final concentration of 1 μg/ml. Such samples were incubated for 30 min at 37°C with the conjugation product of mVirD2-GFP with the MOSIC produced aptamer HeA2_3 (32). For the dot blot experiment, 2.5 × 10^6^ cells were lysed on ice with PBS containing 1% Triton X-100, 5 10^−4^ M PMSF and 0.02% NaN_3_ for 30min and centrifuged at 13000 xg at 4°C for 5 minutes. The supernatant was stored at -80°C until used. A methanol activated PVDF membrane (Biorad) was washed in TBS 1x and spotted with 2 ul of the lysate in a five-fold serial dilution. A 1 mg/ml BSA solution was used as negative control. The membrane was blocked overnight at 4°C without shaking with a 1% western blot blocking solution (Roche) in TBS 1×. The membrane is then incubated at room temperature for 2 h with the mVirD2-GFP-HeA2_3 aptamer in TBS 1× with 0.5% western blot blocking solution (Roche). The membrane was washed three times every 5 min with TBST and imaged for the GFP signal.

### Transient expression of mVirD2 displayed on cell surface and characterization

The coding sequence of mVirD2 was enriched by PCR with an IgG secretion tag and the transmembrane domain (TM) of PDGFRB (Uniprot: P09619) and cloned in pEGFP-NI under the control of a CMV promoter. On the first day, HEK293T cells (550.000/well) were seeded in a six-well plate and cultured at 37°C 5% CO_2_ atmosphere in DMEM/GlutaMAX (Gibco) supplemented with 10% FBS (Sigma Aldrich), 1% Pen/Strep (Sigma Aldrich). The next day, 2 ug/well of the purified plasmid were used for transfection with the Universal Transfection Reagent (Sigma Aldrich) according to the manufacturer's guidelines. Twenty four hours later, transfected and non-transfected cells were incubated for 2 h at 37°C with 1 μM phosphorothioate modified T1 oligo (Cy5 labeled). Cells were dissociated using either a Trypsin/EDTA solution (Thermo Fisher) or the non-enzymatic dissociation solution (Sigma-Aldrich), washed with PBS prior analysis on a FACSAria III Cell Sorter system (BD).

### Generation of a synthetic transcription activator

Under the control of a CMV promoter the mVirD2 coding sequence was fused with the previously characterized synthetic transactivator VPR (VP64-P65-RTA). HEK293T cells (75 000/well) were seeded in a 24-well plate, cultured and transiently transfected as before. As reporter, a PCR fragment enclosing a target site (CD47 promoter), the minimal thymidine kinase promoter from herpes simplex virus, the nanoluciferase coding sequence and the polyadenelation sequence of the soluble neuropiline-1 was transfected the next day alone or pre-incubated with a polypurine reverse Hoogsteen hairpin hosting the mVirD2 target sequence. The fourth day collected cells were lysed in ice with 100 ul of triton X-100 (Sigma-Aldrich) 0.1% in PBS buffer. The luminescence is recorded mixing in equal amount the lysate with the nanoGLO substrate (Promega) in a Varioskan Lux plate reader (ThermoFisher).

### Image acquisition and data plotting

All the gel and dot blot pictures have been acquired on a LAS 4000 imager (GE). Gel bands were quantified on the raw picture with ImageJ software and data was plotted with Graphpad prism software. Microscopy was performed on a Nikon Eclipse Ti inverted fluorescence microscope using a CFI plan apochromat Lambda 40× air objective. Cells were imaged in widefield fluorescence mode using lasers with wavelengths 405 nm (300 mW) and 488 nm (200 mW). Fluorescence emission was detected using ET450/50m and ET525/50m filters and an EMCCD camera: Andor iXon Ultra 888 (1024 × 1024 sensor size, 13 μm pixel size).

## RESULTS

### Expression, purification and validation of a novel self-tagging domain

The novel self-tagging domain herein proposed (mVirD2) has been inspired by the relaxases domain (from aa 1 to aa 204) of the *A. tumefaciens* protein VirD2 from pSDM3149 (17). According to the data we present here, this 24.5 kDa domain clearly retains the ability to covalently bind a specific target sequence through a transesterification reaction (10), just as in the case of the full-length protein. The coding sequence of mVirD2 was amplified by PCR using primers that enabled the incorporation of an affinity tag (6xHis) encoding sequence at the 5′ and a terminator sequence at the 3′ end. The construct was cloned under an IPTG inducible T7 promoter. The protein was overexpressed in BL21 (DE3) T1R pRARE2 cells and purified with high yield (Supplementary Figure S1).

The purified self-tagging domain mVirD2 was eluted in TKM buffer, which also provides the amount of magnesium (1 mM) required for the conjugation reaction to take place (16). Its site-specific conjugative activity was assessed in vitro using fluorescently labeled ONs in a gel mobility shift assay (EMSA) (Figure 1C). mVirD2 was incubated for 1 h at 37°C with a 3′ Cy3-labeled ON containing the binding (square brackets) and cleavage site (∧) of pTiA6 right border (33) (T1 oligo: GCTCAAATTA[CAACGGTATATATCCTG∧CCA]GTCAG-Cy3). The same sequence was previously used to characterize the activity of the purified wild-type protein (16). In a 10% native PAGE gel, the formation of the expected conjugation product is observed as a delayed Cy3 and SYBR gold labeled band, confirming that the purified domain is active (Supplementary Figure S2). Moreover, as further evidence of the conjugation reaction, the cleaved 5′ extremity of the oligo becomes visible only after SYBR gold staining. This portion of the ON T1 (28 bases) contains the binding site of mVirD2 being SYBR gold-positive but Cy3-negative and it gets detached from the 3′ extremity when the tyrosine 29 in the catalytic site forms the ester bond at the cleavage position in the oligo sequence (34). One advantage of this self-tagging domain is that most of the consensus sequence required for the conjugation is removed by nicking, leaving only three bases upstream the sequence of interest (i.e. leaving greater flexibility for freely choosing the conjugate oligo sequence).

To determine the conjugation reaction rate, fixed amounts of mVirD2 and T1 ON, respectively 250 ng and 25 pmole, were incubated in 10 μl TKM buffer in a thermocycler at 37°C for increasing amount of time (from 30 min to 4 h). The reactions were stopped at the same moment and checked by EMSA in a 10% native PAGE. Despite a continuous signal increase with time from the DNA–protein complex (detected trough the Cy3 channel), the intensity of the ON 5′ nicked product band revealed by SYBR gold post-staining, is rather stable already after 1 hour of incubation and does not significantly increase over time (Supplementary Figure S3A). As for other relaxases, the conjugation of mVirD2 is a two-step reaction where the nicking of the target DNA follows the target sequence binding reaction that appears to be faster (35). Subsequently, increasing amount of the T1 ON (from 0.39 to 50 pmol) were incubated with mVirD2 (250 ng) for 1 h at 37°C in a 10 μl reaction volume of TKM buffer leading to the determination of the apparent *K*_d_ value of 468 ± 68 nM by EMSA with a 10% native PAGE gel (Supplementary Figure S3B). These values are of the same order of magnitude as for other relaxases proteins (19). Furthermore, Coomassie staining of the same gel enables to detect the presence of the protein in the complex while unconjugated proteins migrate poorly in the gel due to the gel conditions favoring DNA migration.

The conjugation yield approaches 100% with regards to the DNA if a large excess of protein is available (Supplementary Figure S3B). However, the yield with regard to the fraction of protein that is labeled appears to saturate at ∼40% (Supplementary Figure S3c). The equilibrium of the reaction is rapidly reached because the nicked fragment is available for re-ligation. To remove eventually unconjugated ONs or nicked fragments from the reaction, we optimized an enzymatic clean-up (Supplementary Figure S9). Exonucleases with 5′ to 3′ polarity have the ability to digest all nucleic acids made exception for those conjugated that are 5′ protected by mVirD2. It has been shown that, *in vitro*, the protein–DNA bond is stable once the nicked fragment is purified away (44). It was previously demonstrated that phosphorothioate modified ONs (PS-ON), also called suicide ONs, synthetized with substitution of a non-bridging oxygen of the phosphate for a sulfur, in addition of showing increased resistance to nucleases (36), would also enable an irreversible conjugation rather than a dynamic conjugative equilibrium (19,37). Using a T1 PS-ON, it was possible to observe an extensive conjugate yield increment also for mVirD2 (Supplementary Figure S4). Even if the aim of the current work is to present a method for a complete enzymatic production of conjugates such ON modification could be of interest for other applications, like the ability to display DNA on cell membranes or control the gene expression using a synthetic transcription activator, where it is possible to introduce ON from outside and high conjugation yield is a paramount.

In previous studies, it has been shown that a very similar mechanism of bond formation via transesterification, could also lead to transient protein–RNA interactions (38,39). We checked if mVirD2 would also be able to bind RNA. Despite the fact that a preliminary *in-silico* domain homology based screening was in favor of this hypothesis, the gel shift assay with synthetic RNA and DNA-RNA chimeric ONs demonstrated that mVirD2 is able to bind the target ON only if the AT-rich binding sequence is made of DNA, whatever the target sequence was single or double stranded. We were not able to appreciate any nicking of ONs with RNA chemistry (Supplementary Figure S6).

To prove that mVirD2 can act as a self-tagging domain, a fusion protein was generated. The coding sequence of a flexible linker (GGSGGGSG) and emGFP were inserted by PCR at the C-term of mVirD2 (Supplementary Information 1). The fusion protein (52 kDa) was overexpressed and purified as described before. We observed that a very small fraction of the purified protein is lacking the GFP, the premature truncation could be induced by the mRNA structure at the hinge between mVirD2 and emGFP. To verify if the fusion product disturbs the self-tagging domain, mVirD2 activity was compared using the Cy3-labeled ON T1 (Figure 1C). The collected evidence from the EMSA gel revels a comparable conjugative activity. Moreover, a precise quantification of the band intensity of the nicked product from the post-stained gel with SYBR gold (Supplementary Information 5) showed a similar saturation trend as mVirD2 alone (Figure 1B). Thus, it is possible to confirm that fusion constructs with mVirD2 form suitable tags for ONs bioconjugation.

### Enzymatic ONs synthesis (MOSIC) and targeted conjugation in a one-pot reaction

To overcome the limitations of the solid state ONs synthesis such as yield and length (40,41), we earlier demonstrated that it is possible to efficiently produce considerable amounts of high quality ONs using an enzymatic strategy (22). Herein, we apply the ability of the novel self-tagging mVirD2 domain to efficiently bind its target under the conditions used in the protocol for the enzymatic production of ONs. We observed that mVirD2 activity is not affected by the composition of the different reaction buffer (Supplementary Figure S7).

**Figure 2.**
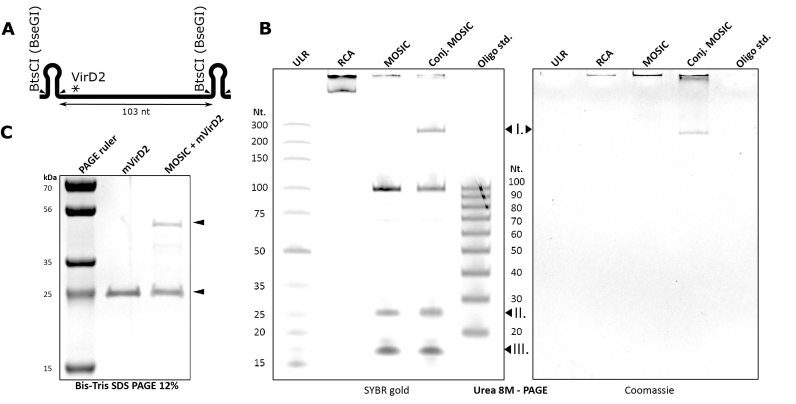
Enzymatic oligonucleotide production and conjugation in a one one-pot reaction. (**A**) A pseudogene, enclosing the sequence of an ON of interest is designed. A unique binding site for mVirD2 (*) is fused at the 5′ of the HeA2_3 aptamer sequence placed between two hairpins that enable the digestion of the rolling circle amplification product. The pseudogene is re-circularized and used as template for the MOSIC protocol. The amplification product of the pseudogene (RCA), the hairpin digestion product (MOSIC) and the conjugation product (Conj. MOSIC) were assessed in a denaturing PAGE gel (**B**). High molecular weight RCA product concatamer are clearly unable to migrate in the gel while once the hairpins are digested the oligonucleotide of interest is revealed, 103 nucleotides, together with the hairpin (**II**.) and inter-hairpin sequence (**III**.). The covalent mVirD2-oligonucleotide conjugation product band (**I**.) is clearly delayed in the migration. Overlapping signal of the conjugate after proteins staining with Coomassie. ULR: ultralow range ladder (Thermo Fisher), Oligo std.: ssDNA ladder 20–100 nucleotides (IDT). (**C**) The conjugation reaction is also evident in a highly resolving protein gel, 12% Bis-Tris SDS-PAGE, stained for total protein with Coomassie. Here mVirD2 (25 kDa) before and after the conjugation reaction is loaded.

To exemplify this combined production and conjugation protocol, a pseudogene hosting a 103 nucleotides ON, including the 17 nucleotides long mVirD2 consensus sequence (Figure 2A and sequence in Supplementary Information 8), was designed and processed as a template for the MOSIC production of ONs (22,23). The quality of the product was assessed by denaturing gel electrophoresis and the MOSIC product was combined with mVirD2 (500 ng) and incubated for 1 h at 37°C. The equivalent of 1 μl of RCA amplification, of digested RCA (i.e. MOSIC product) and of conjugation reaction were incubated with 1 volume of 90% formamide, 0.5% EDTA and 1% Orange G solution at 70°C for 15 min and directly loaded in a 10% 8 M Urea PAGE (Figure 2B). As expected, we observed a poor migration of the RCA concatamer while the complete digestion of the RCA product revealed the designed ON (103 nucleotides) and the digestion byproducts (**II**. and **III**.). A considerable amount of the oligo (**I**.) is delayed in the migration as consequence of the conjugation with the self-tagging domain. The presence of the mVirD2 tag was confirmed by successive colloidal Coomassie staining. The full reaction was conducted with no buffer exchange from beginning to end and the conditions are mild enough that it should avoid stress to most proteins. To give a clearer picture of the reaction, we also depleted the MOSIC product from its proteins and incubated with 500 ng of mVirD2 in same conditions as above (Figure 2C). These samples were then denatured at 95°C for 5 min in Laemmli buffer and loaded in 12% Bis-Tris PAGE. Even if such staining lacks of signal linearity for quantification, the resolving power of this gel enabled us to distinguish the free protein from the conjugate product (arrowheads).

### Simultaneous site-specific conjugation of multiple target ONs

**Figure 3.**
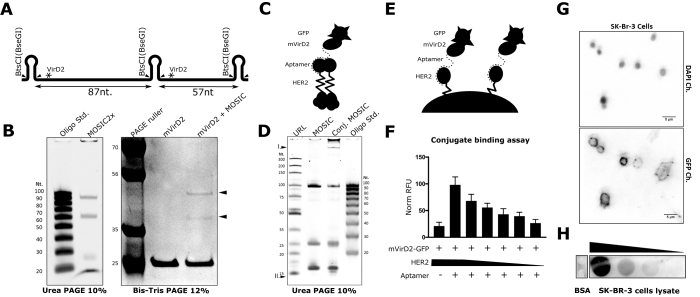
Production and conjugation of multiple ONs and application of a conjugate as biomarker detector. (**A**) Schematic representation of a pseudogene (MOSIC2x) that enables the enzymatic production of two short ONs, respectively 87 and 57 nucleotides, each with a binding site for mVirD2 (*). The quality of the MOSIC digestion is first assessed in a 10% urea–PAGE gel. Oligo std.: ssDNA ladder 20–100 nucleotides (IDT). Highly resolving denaturing 12% Bis-Tris SDS-PAGE, stained with Coomassie (**B**), shows the formation of two delayed bands (arrowheads) after the conjugation reaction. PAGEruler plus ladder, Thermo Fischer. A cancer biomarker detection device was entirely enzymatically assembled (**C**) and controlled in a denaturing PAGE gel (**D**). The conjugate is considerably delayed in the gel (**DI**) and the nicked product is visible after conjugation (**DII**). The ability of mVirD2-GFP-HeA2_3 aptamer conjugate to bind is target was assessed *in-vitro*, immobilizing the recombinant protein on magnetic beads (**E**). A constant amount of the conjugate was incubated with decreasing amount of the target protein and the GFP fluorescence was recorded (**F**). The conjugate was used to detect HER2 protein in fixed cells (**G**) and in cell lysate (**H**).

As a proof of concept of the freedom degree offered by this protocol concerning the length and number of ONs that can be produced, we decided to design a second pseudogene able to generate two short ONs, respectively 87 and 57 nucleotides (Figure 3A and sequence in Supplementary Information 8). The same protocol enabled the facile production of the two oligonucleotides from the same pseudogene (MOSIC2x) as showed in the urea–PAGE and to conjugate them both due to the presence of the 17 nucleotides consensus sequence (Figure 3B).

### Production of a GFP-tagged aptamer

We took advantage of this protocol to produce a GFP conjugate of a previously described aptamer for the membrane protein HER2 (32) (Figure 3C). The fabrication of this detection device for an important cancer biomarker was fully enzymatic. Using this technique could reduce costs and enable larger scale screening of aptamers without the use of fluorophore-modified oligos. The ON and the conjugate quality were evaluated with the help of a denaturing gel (Figure 3D). The ability of the ON-protein complex to bind its target was first assessed *in-vitro* using dynabeads (Figure 3E and F). Such entirely enzymatically produced conjugates were successfully used to stain cells known to strongly express HER2, and also to detect the protein in cellular lysates in a dot blot (Figure 3G and H).

### Cell membrane labeling and oligonucleotide mediated transcription activation

As a proof of concept of the compatibility of mVirD2 in biological systems and to broaden possible applications of the new self-labeling tag, two new constructs were generated.

**Figure 4.**
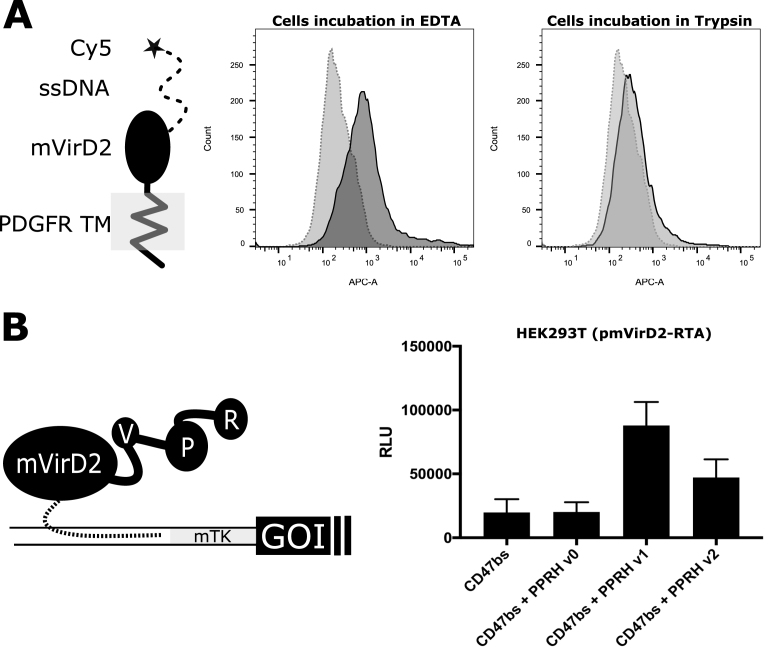
Expression of a transmembrane mVirD2 protein for cell membrane labeling and construction of a novel oligonucleotide-mediated trans-activator. (**A**) Schematic representation of the chimeric protein bound to a Cy5 labeled oligonucleotide and FACS distribution of Cy5 labeled cells. The plots enclose the profile given by untransfected cells, incubated with the oligonucleotide (light gray) and transfected cells incubated with the oligonucleotide (dark gray). After labeling, the cells were detached from the surface chemically (EDTA) or enzymatically (Trypsin) and washed with PBS prior FACS analysis. (**B**) Representation of the synthetic transactivator (V: VP64, P: P65, R: RTA) in connection with the reporter cassette through mVirD2 and an oligonucleotide upstream a gene of interest (GOI). On the right, protein normalized relative luminescence given by the reporter construct (olifonucleotide target site, thymidine kinase minimal promoter and nanoluciferase coding sequence). HEK293T cells transiently expressing mVirD2-RTA were transfected again with the reporter construct alone (CD47bs) or together with an oligonucleotide. PPRH v0 is designed to bind a specific domain upstream the minimal promoter (but without the mVirD2 consensus sequence) while PPRH v1 and v2 also host the mVirD2 consensus sequence in connection with two linkers of different lengths. When such ON are co-transfected they enable a considerable luminescence increase.

The first one, pmVirD2-TM enabled the expression of a membrane displayed mVirD2 (Figure 4A). Just the addition of oligonucleotides in the media enabled an effective labeling of the cells expressing the transmembrane (TM) protein. We earlier (Supplementary Figure S9) validated that the conjugate can be disrupted by the proteolytic activity of trypsin and used such approach to verify the nature of the membrane labeling. The FACS histograms highlight the difference between the cell dissociation procedures (dark gray).

The second construct, mVirD2-RTA, enabled the expression of a novel transactivator fused at the C terminal of mVirD2 (Figure 4B). The aim of such chimeric protein, inspired by previous work (42), is to control the expression of a gene of interest by bridging the transcription activator with the help of an ON. The used ON (dotted line) is a characterized polypurine reverse Hoogsteen hairpin (PPRH v0) that specifically targets the promoter region sequence of CD47 (43) and was fused with the PS modified consensus sequence for the conjugation to mVirD2 (Supplementary Information 10). A reporter construct (CD47bs) encoding the nanoluciferase gene was used to validate the activity of this transactivator in HEK 293T cells earlier transfected with mVirD2-RTA. A consistent overexpression of the reporter was recorded in presence of fused PPRH to mVirD2 consensus trough a (T)_5_ linker (PPRH v1), while the C18 linker (PPRH v2) reduced the efficacy.

## DISCUSSION

In this study, we have shown that the minimal version of VirD2 retains the self-tagging properties of the full-length protein. We thus demonstrate that this domain can enable an enzymatic, covalent and site specific oligonucleotide conjugation. Additionally, protein fusion to this mVirD2 does not hinder that function in our example applications. Notably though, this does require a case-by-case assessment, similar to any other conjugation method. In our hands, mVirD2 seems to not be able to nick and conjugate to RNA. More data is however needed to conclude whether this could be a general trait common in most DNA relaxases. Moreover, our data shows that these protein constructs can be used in a convenient one-pot assembly together with our previously reported MOSIC method for enzymatic production of single-stranded oligonucleotides. The method presented here thus represents, to our knowledge, the first completely enzymatic DNA–protein conjugation strategy that can be used for biological production of short ssDNA ONs covalently attached to proteins. Using these conjugates, we were able to produce a highly active staining reagent for HER2, label cell membranes and control the expression of a reporter gene by simple addition of the reactive ssDNA. Even if such a method presents some intrinsic limitations, such as forced directionality of the conjugated oligonucleotide as well as the need for genetic manipulation, starting from biologically produced components could be important in many applications. In contrast to all other protein tagging methods, our method does not require any synthetic chemistry, neither on the DNA nor on the protein, and could therefore conceivably open a path to a complete production of DNA–protein conjugates directly from lysates, directly in bacteria or possibly even in-vivo.

## ACCESSION NUMBERS

Complete sequences of the plasmids pmVirD2 (ID: 90314) and pmVirD2-GFP (ID: 90315) are available at Addgene.org.

## Supplementary Material

Supplementary Data
