# Supplementary material for: Entirely enzymatic nanofabrication of DNA–protein conjugates
Source: Nucleic Acids Res. 2017 Aug 16;45(18):e160. doi: 10.1093/nar/gkx707 (PMC5737863; doi:10.1093/nar/gkx707)
Supplement: Supplementary Data [file gkx707_supp.pdf]

Entirely enzymatic nanofabrication of DNA-protein conjugates

Giulio Bernardinelli & Björn Högberg

MBB, Karolinska Institutet, 17 177 Stockholm, Sweden

Supporting information

(S1) Constructs cloning and proteins purification.

The coding sequence of **mVirD2** (24,5 kDa) was amplified by PCR from pSDM3149 using primers that enabled the insertion of a 5' 6xHis tag and a 3' terminator. The PCR product was purified from a 1% Agarose gel (TBE 0.5X, 100V 30 min) and cloned in pSNAP-tag (T7)-2 (NEB) as described in the materials and methods section. Further sequence details available on Addgene.org (plasmid: **90314**)

```
> [p-mVirD2.xdna]
GATATCattaaagaggagaaaACTAGTATGcatcatcatcatcatcatCCCGATCGCGCTCAAGTAATCATTCGCATTGT
GCCAGGAGGTGGAACCAAGACCCTTCAGCAGATAATCAATCAGTTGGAGTACCTGTCCCGTAAGGGAAAGCTGGAACTGC
AGCGTTCAGCCCGGCATCTCGATATTCCCGTTCCGCCGGATCAAATCCGTGAGCTTGCCCAAAGCTGGGTTACGGAGGCC
GGGATTTATGACGAAAGTCAGTCAGACGATGATAGGCAACAAGACTTAACAACACACATTATTGTAAGCTTCCCCGCAGG
TACCGACCAAACCGCAGCTTATGAAGCCAGCCGGGAATGGGCAGCCGAGATGTTTGGGTCAGGATACGGGGGTGGCCGCT
ATAACTATCTGACAGCCTACCACGTCGACCGCGATCATCCACATTTACATGTCGTGGTCAATCGTCGGGAACTTCTGGGG
CACGGGTGGCTGAAAATATCCAGGCGCCATCCCCAGCTGAATTATGACGGCTTACGGAAAAAGATGGCAGAGATTTCACT
TCGTCACGGCATAGTCCTGGATGCGACTTCGCGAGCAGAAAGGGGAATAGCAGAGCGACCAATCACATATGCTGAACATC
GCCGCCTTGAGCGGATGCAGccatggTAAcgcaaaaaaccccgcttcggcggggtttttcgcggatccccc
```

The coding sequence of the fusion product **mVirD2-GFP** (51,8 kDa) was obtained by overlap extension PCR as previously described. In first instance the following primers (GFP_FW, GFP_REV) were used to amplify emGFP. This first cycle enabled the insertion of a 5' flexible linker. The 776bp product is amplified again by a second PCR (primers ExtPCR1, ExtPCR2) to introduce homologous sequences for the overlap extension PCR protocol. The product of this second amplification is then used for the overlap extension PCR. Further sequence details available on Addgene.org (plasmid: **90315**)

Oligo name	Sequence 5'->3'
GFP_FW	ggtggttctggtggtggttctggtGTGAGCAAGGGCGAGGAGCT
GFP_Rev	aagtcaaaagcctccggtcggaggcttttgactttttaCTTGTACAGCTCGTCCATGC
ExtPCR1	ACATCGCCGCCTTGAGCGGATGCAGccatgg/ggtggttctggtggtggttctggtGTGA
ExtPCR2	CCTTTCGGGCTTTGTTAGCAGCCGGATCAAT/aagtcaaaagcctccggtcggaggcttttgacttttta

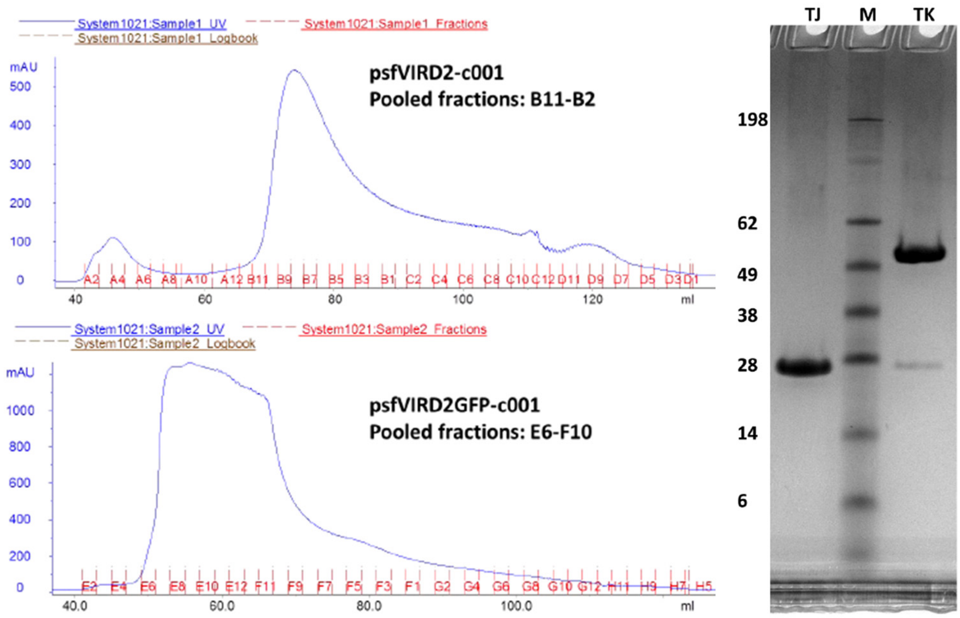

Fig.S1 - Gel filtration elution profiles of each protein and batch analysis of the purified proteins*. TJ: mVirD2, TK: mVirD2-GFP, M: SeeBlue Plus 2 protein ladder (Invitrogen). Both proteins purified well and didn't show any problems with solubility. SDS-PAGE analysis of the VIRD2-GFP additionally showed a protein which is likely mVirD2 without the fused GFP, based on its size. This may be due to incomplete translation of the construct or protease cleavage of the full length protein in the E. coli. Its concentration is however much lower than the full length tagged VIRD2-GFP. The size of the proteins was further verified by mass spectroscopy resulting 24589.35 Da for mVirD2 and 51864.91 Da for mVirD2-GFP.*

(S2) mVirD2 in-vitro activity scheme:

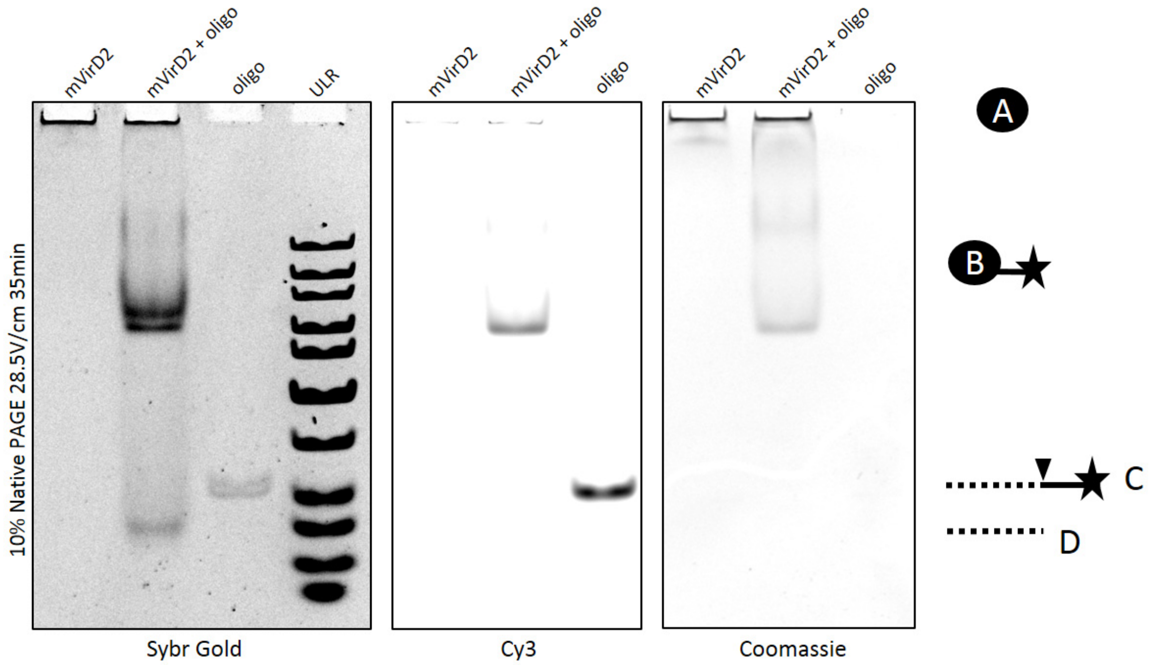

Fig.S2 - Conjugation reaction between the minimal relaxases (mVirD2) and a Cy3 labeled ONs (T1) that contains the consensus sequence*. The reaction was assessed in a 10% native PAGE gel. ULR: ultralow range ladder (Thermo Fisher). The ON T1 (oligo) (**C**) is site-specifically nicked (arrowhead) and the protein establishes a bond with the Cy3-labeled fragment (**B**) while the nicked fragment (**D**) migrates faster. As revealed by protein staining (Coomassie), mVirD2 (**A**) poorly migrate in the gel unless it is coupled with the DNA molecule.*

(S3) Conjugation reaction characterization

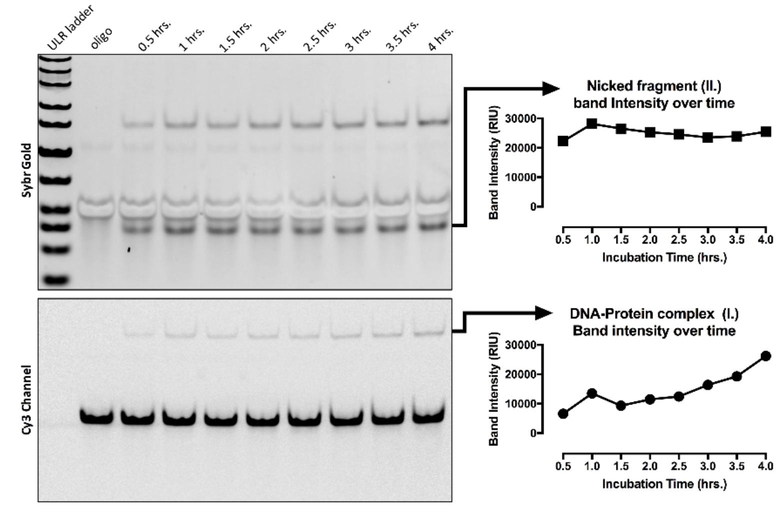

***Fig.S3a - mVirD2 conjugation reaction rate**. To determine the optimal incubation time for the conjugation reaction, a set of 8 identical reaction mix (250 ng mVirD2, 25 pmol of labeled oligonucleotide in 10 µl TKM buffer) were incubated at 37°C for increasing amount of time (from 30 min. to 4 hrs.) and checked with a 10% native PAGE. Despite a continuous signal increase of the DNA-protein complex (delayed band), detected trough the Cy3 channel (**I.**), the amount of the DNA digestion product, revealed by Sybrgold post-staining only (**II.**), is relatively stable after 1h of incubation. The intensity of the bands (**I.** and **II.**) was measured with ImageJ software and plotted aside.*

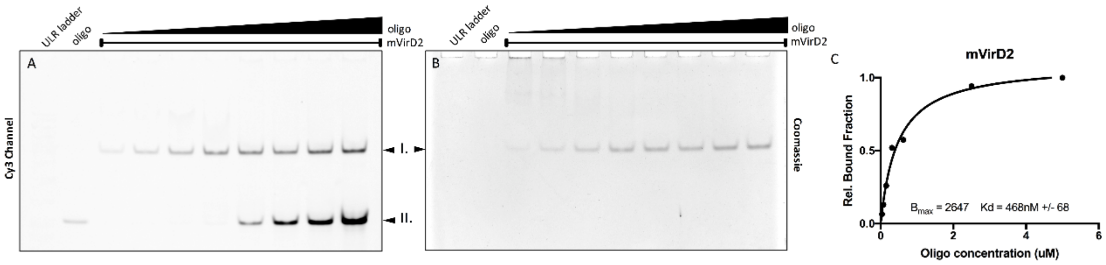

***Fig.S3b – Measurement of the apparent Kd of mVirD2.** A constant amount of mVirD2 protein (250 ng) has been incubated with a doubling amount of target oligonucleotide T1 (from 0,39 pmol to 50 pmol in a 10 µl reaction in TKM buffer). The Samples, including the oligonucleotide without the protein were incubated at 37°C for 1 hour prior electrophoresis in a 10% native PAGE. The same gel has been (**A**) imaged for Cy3 signal and (**B**) post-stained for the total proteins with colloidal Coomassie. The apparent Kd measurement were conducted as previously reported (19). The fraction of bound Oligonucleotide (**I.**) has been measured with ImageJ and plotted (**C**) as fraction of maximum binding recorded. The obtained values were graphed with Graphpad Prism software and fitted using a one site specific binding hyperbola. It is possible to observe a clear correspondence of the bound oligo with the mVirD2 protein*

*signal as well as an increase of the proportion of protein able to migrate in the gel with the increase amount of oligo in the reaction (**I.**). While the excess unbound oligo (**II.**) migrate regularly.*

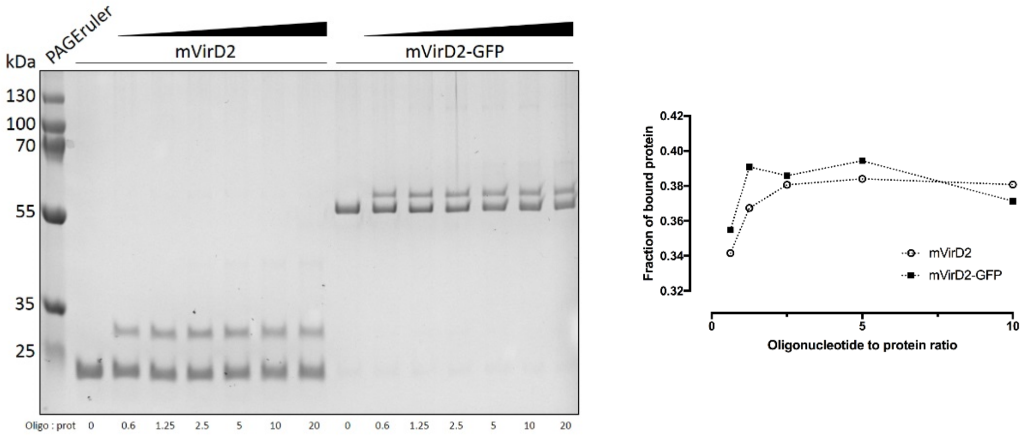

Fig.S3c – Determination of the fraction of protein conjugated.

To determine the amount of protein covalently linked to the oligonucleotide the conjugation reaction (10 µl) was assessed in 12% Bis-tris PAGE and stained as previously described. The protein (mVirD2 or mVirD2-GFP) were incubated with an incremental excess of the oligonucleotide (molar ration from 0.625:1 to 20:1 oligonucleotide: protein). The intensity of the delayed band (conjugation product) was quantified with ImageJ and graphed aside. The binding capacity of the enzyme is not affected by the fusion product and the binding capacity is rapidly reached.

(S4) Effect of Phosphorothioate modified oligonucleotides on the conjugation reaction.

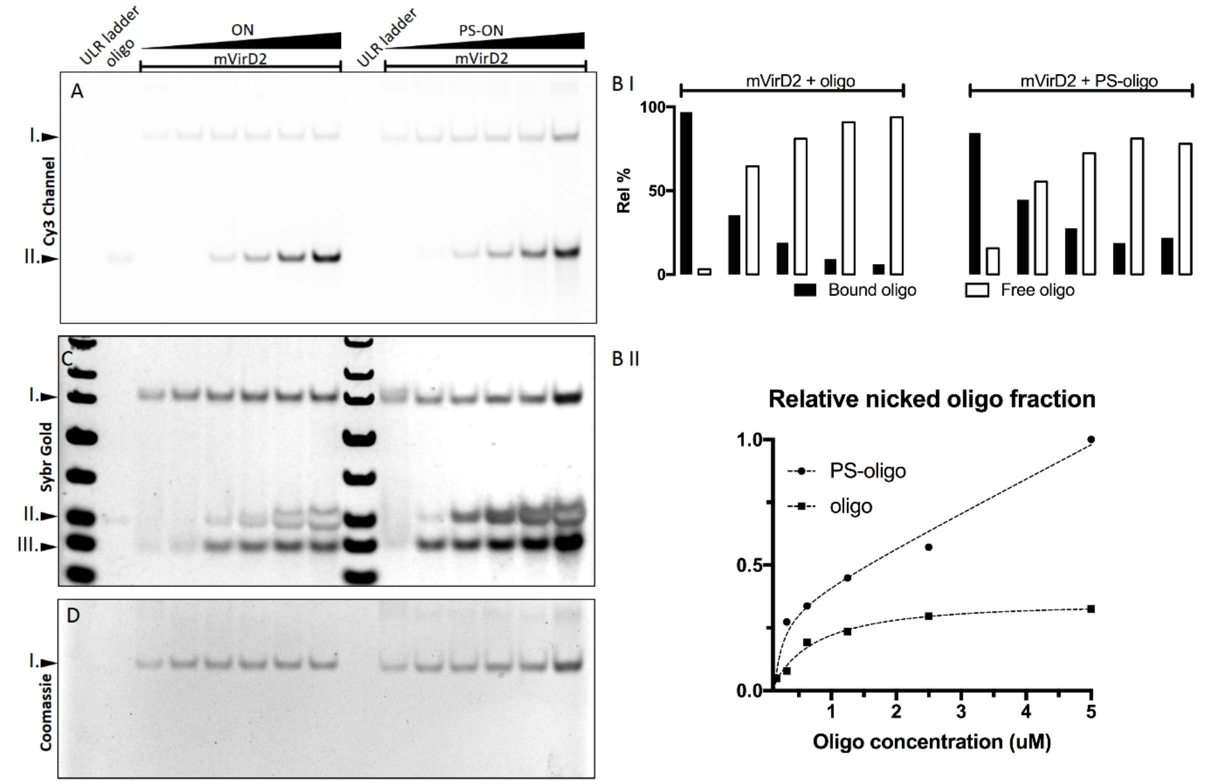

Fig.s4 - Phosphorothioate modified oligonucleotides increase considerably the conjugation yield. *Herein, six 10 µl reactions containing 250 ng of mVirD2 and a 2-fold ON serial dilution (from 50 to 1,5 pmol) containing either the Cy3-labeled unmodified or PS-modified ON (see table below) in TKM buffer, were ran in a 10% native PAGE after an incubation at 37°C for 1 hour.*

*The same gel has been imaged first for the Cy3 channel (**A**) then for the total nucleic acids with sybrgold (**C**) and finally for the total proteins (**D**). The arrowheads indicate (**I.**) the DNA-protein complex, (**II.**) the free oligo and (**III.**) the nicking product. The Relative amount (obtained from the Cy3 channel) of free and bound oligonucleotide of each reaction is showed in the bar graph (**B I**). The cumulative amount of digested (nicked fragment, gel **C**) ON is plotted in the second graph (**B II**). It is then possible to confirm that such modification also improves the conjugation reaction for mVirD2 and it has not been possible to observe a saturation of the reaction.*

Oligo Name	Seq 5'-> 3' Nick site: ^	Notes
**T1** (VirTarget)	GCTCAAATTACAACGGTATATATCCTG^**CCAGTCAG(Cy3)**	Ref. Ziemienwicz et al.
**PS-T1**VirTarget	GCTCAAATTACAACGGTATATATC**<u>CTG^CCA</u>GTCAG(Cy3)**	Underlined bases linked by PS bond

(S5) Figure 1 with SYBR gold staining

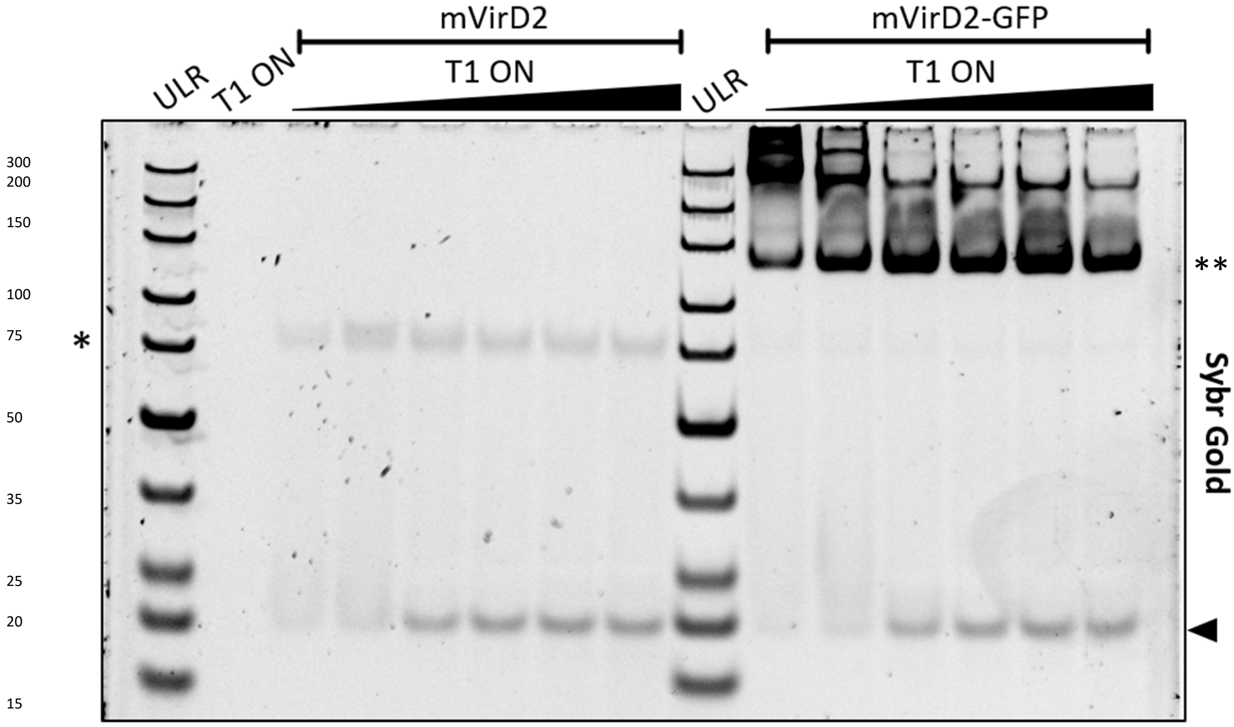

***Fig.S5 – Activity comparison of mVirD2 and mVirD2-GFP**. The same gel in **figure 1** was also stained for total nucleic acids with Sybergold. In here it is possible to observe the DNA associated with mVirD2 (*) and how the migration of mVirD2-GFP, overlapping emission spectrum, is transitioning toward the conjugate thus more mobile in the electric field (**). Moreover, the signal from the nicked 5' segment (arrowhead) was quantified with ImageJ and used to quantify the Relative nicked oligo fraction in the graph of **figure 1B**. ULR: Ultra low range ladder 300-15 (ThermoFisher).*

(S6) mVirD2 does not interact with RNA

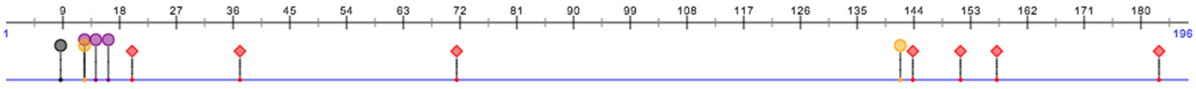

In silico prediction of a RNA binding motif: Output prediction from https://www.predictprotein.org/
RNA-like binding region SO:0000934: A miRNA target site is a binding site where the molecule is a micro RNA. Parent: nucleotide_binding_site (SO:0001655)" possible amino acids involved: 14-22, 16, 18.

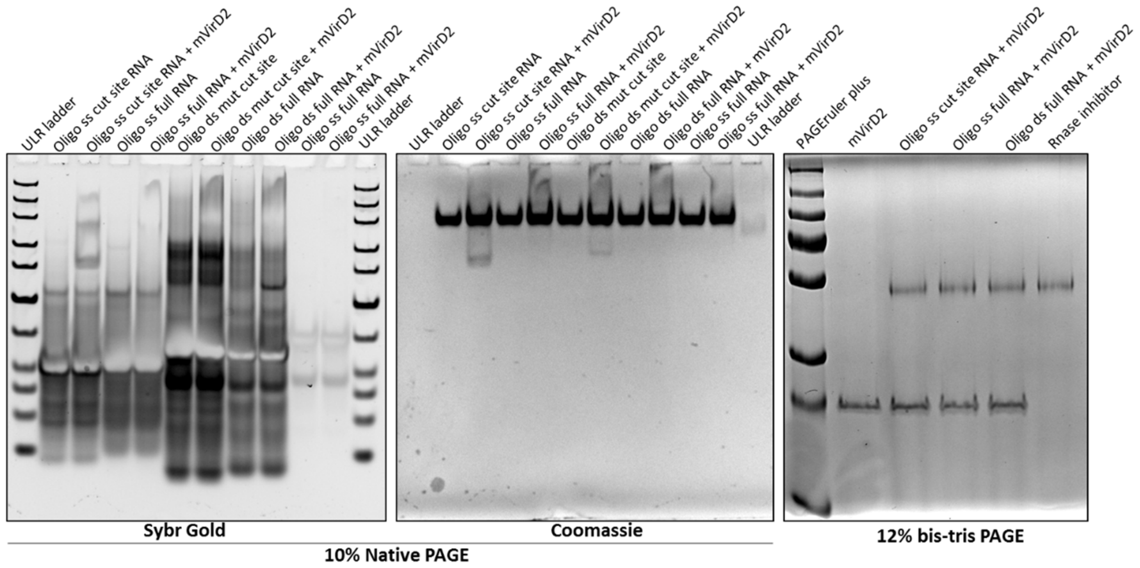

Fig.S6 - mVirD2 is unable to conjugate to RNA molecules*. EMSA experiment in a 10% native polyacrylamide gel. The multiple secondary conformation of oligonucleotides probabely determined a smear in the gel. All the sample (10 µl) were incubated at 37°C for 1 hour in presence of murine RNase inhibitors, 4000U, (NEB) with or witouth mVirD2. A major shift is observed only when mVirD2 is incubated with "ss Cut site RNA" where the AT rich consensus sequence is deoxiribonucleotide while, when the binding site is made of ribonucleotide no retardation is observed. Surprisingly a weak oligonucleotide-protein interacction is observed with a mutated consensus sequence, higlighted in the Coomassie staining. As no band retardation is observed in the Bis-Tris denaturing 12% PAGE it possible to assume that even if some interactions are observed, such are not of covalent nature. Moreover, the construction of ds RNA does not help the conjugation reaction.*

Oligo name	*Oligo sequence 5'*
ss Cut site RNA	GCTCAAATTACAACGGTATATATCCT**GCCA**GTCAG
ss full RNA	G**GGAACACUACUAAACUUCAUAUAUCCUGCCAGCCAAUCAAAUCU**
ds mut cut site	TTTATTTGTCTCTGAGGGCTCTACGGAGTCTT**CAGAGACAAAUAAAU**GCCCGTCCGGGC
ds full RNA	**GGAUGUAUGAAGUCGAUCCGUCGGCUUCAUAUAUCCUGCCAGGAGCAUACGUGCUCUUGGUA**

bold: Ribonucleotide base, ss: single stranded, ds: the oligo is predicted to fold uppon itself and form a dumbell shape. Mut: consensus sequence mutated.

(S7) mVirD2 is active in RCA buffer

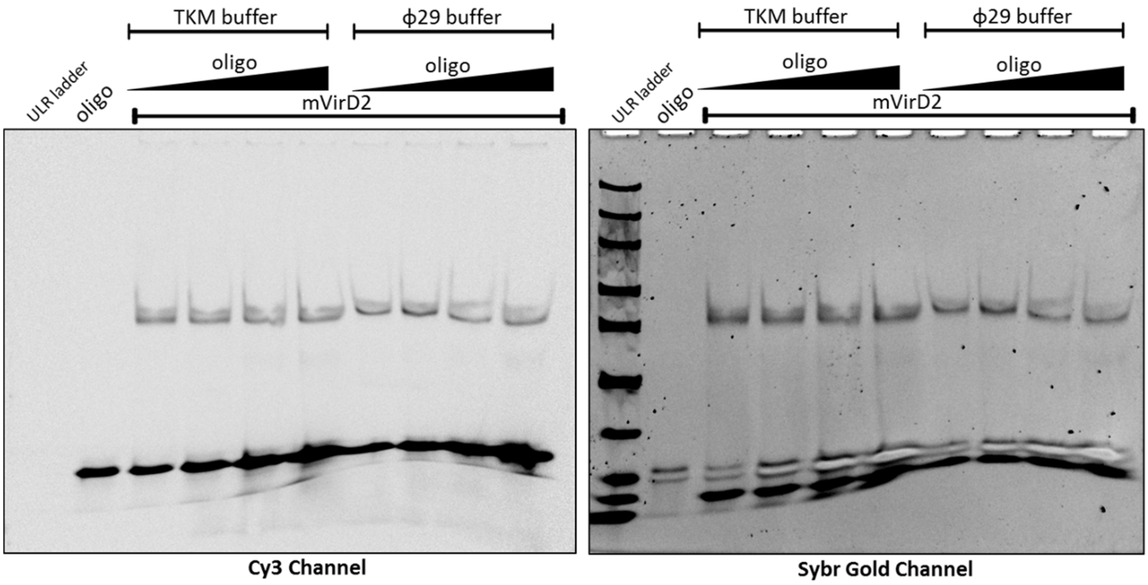

Fig.S7 *– EMSA 10% native PAGE of a conjugation reaction of mVirD2 (250 ng) with T1 ON (50 to 6,25 pmol) in regular TKM or in the Phi29 polymerase buffer (33 mM Tris-acetate pH 7.9, 10 mM Mg-acetate, 66 mM K-acetate, 1 mM DTT) for 1 hour at 37°C. Even if the salt difference of the buffers generated a distortion in the electrophoretic migration it is possible to conclude that as magnesium is present in the phi29 buffer enable a normal activity of mVirD2.*

(S8) MOSIC pseudogenes sequences

MOSIC Pseudogene_1x for the enzymatic production of the sequence HeA2_3:

GCTCAAATTACAACGGTATATATCCTGCCAGTCAGTAGGGAAGAGAAGGACATATGATTCTAAAAGGATTCTTCCCAAGGGG ATCCAATTCAAACAGC.

> [ConjMOSIC_1x.gb.xdna - 188 bp]
GGTCTCACATTGCATAAGCTTCATCCGTGGGAACCACGGATGAAGCTCAAATTACAACGGTATATATCCTG^CCA*GTCAGTAG GGAAGAGAAGGACATATGAT***TCTAAAAGGATTCTTCCCAAGGGGATCCAATTCAAACAGC**CATCCGTGGGAACCACGGATGGC TGGCTCTTCATGCATTGGAGACC

(MOSIC hairpins; ^: nicking site; *linker*; **HeA2_3 aptamer**)

Mosic Pseudogene_2x:

> [conjMOSIC2x.gb.xdna - 277 bp]
CTCGAGGGTCTCACATTGCATAATTTTCATCCGTGGGAACCACGGATGAAAAATTACAACGGTATATATCCTG**^CCAGTCA GCACATCTTCACCCTCACTTCAACTACA**CACCATCCGTGGGAACCACGGATGGTAAATTACAACGGTATATATCCTG**^CCA GTCAGTCTACTTCCACCTCCACTCCCACTCACCTCCACCTCACCCTCACCCTACTTCCACTC**CTCCATCCGTGGGAACCA CGGATGGAGGGCTCTTCATGCATTGGAGACCCTCGAG

(MOSIC hairpins; ^: nicking site; **ONs of interest**)

(S9) Conjugation protects from DNA exonucleases and can be disrupted by proteases

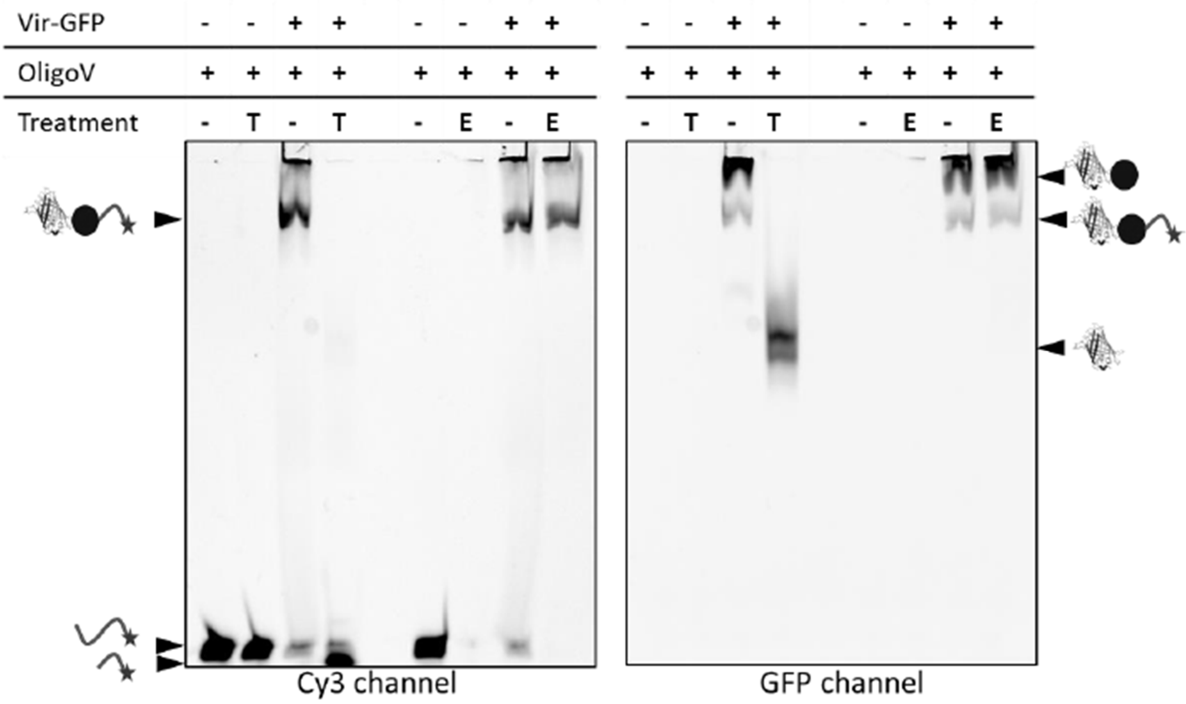

Fig. S9 *– Gel electroforesis (10% PAGE) of the mVirD2-GFP protein-oligonucleotide T1 (3’ Cy3 labeled) complex. Following the Conjugation reaction, the samples were incubated for 30 minutes at 37°C in presence of trypsin (Thermo Fisher) (T) or T5 exonuclease (NEB) (E).*

The conjugated oligonucleotide is notabely protected mVirD2-GFP on the 5’ end from the exonuclease activity while the unconjugated oligonucleotide is almost complitely degradated. On the other hand the trypsin treatment was able to release the T1 oligonucleotide and partially digest the protein.

(S10) Generation of a novel transcription activator and reporter

PVir-RTA: under the control of a CMV promoter the coding sequence of mVirD2 is fused to the chimeric VPR transactivator:

```
(Kozak seq) CGCCACC
(mVirD2) ATGCACCACCACCACCATCATCCCGATCGCGCTCAAGTAATAATCAGAATAGTGCCTGGCGGAGGCACGAAAA
CGCTTCAGCAAATAATTAATCAACTGGAATACCTGAGCCGCAAAGGAAAGCTGGAGCTTCAAAGGTCAGCTCGACACCTC
GACATACCTGTTCCTCCAGACCAAATCCGGGAACTCGCGCAGTCTTGGGTAACAGAAGCGGGCATATACGATGAGTCACA
GTCCGATGACGATAGACAACAGGACCTTACTACACATATTATAGTGTCCTTTCCGGCAGGGACTGACCAAACGGCGGCGT
ACGAGGCATCACGGGAGTGGGCAGCGGAAATGTTTGGATCAGGGTATGGGGGCGGAAGGTATAATTATCTCACAGCCTAT
CACGTCGACCGAGACCATCCCCACCTGCATGTGGTGGTGAACCGAAGGGAGCTGCTGGGACATGGTTGGCTCAAGATTTC
ACGGAGGCACCCCCAGCTCAATTACGACGGACTGCGAAAAAAGATGGCAGAAATTAGCCTGAGACATGGGATTGTGCTCG
ATGCAACATCCAGAGCTGAAAGAGGGATAGCAGAACGCCCAATTACGTATGCGGAGCACAGACGATTGGAGAGGATGCAG
AGCGGAAGT
(VP64)GGGAGGGCGGACGCCCTGGACGACTTCGACCTGGACATGCTGGGCAGCGATGCCTTGGATGATTTCGATCT
CGATATGCTTGGGAGTGATGCGCTTGATGATTTCGACCTCGATATGTTGGGGAGTGACGCTTTGGATGACTTTGACCTGG
ACATGCTG
(VP40 NLS) ATAAATTCCCGCTCTTCAGGTAGTCCGAAAAAAAAACGAAAAGTTGGCAGC
(P65) CAATATCTTCCGGATACGGAC
GACCGCCATAGAATCGAGGAAAAACGGAAACGAACGTACGAGACGTTCAAGAGTATAATGAAGAAGTCACCCTTCTCAGG
ACCTACAGATCCTCGACCCCCGCCTCGCAGAATAGCAGTTCCGAGCCGGAGTAGTGCAAGCGTTCCAAAGCCTGCCCCCC
AACCCTATCCCTTTACTTCTAGCCTCTCTACTATTAACTATGACGAATTCCCCACTATGGTGTTCCCCAGCGGGCAGATA
TCTCAAGCAAGTGCTCTGGCGCCTGCACCACCACAAGTGCTGCCACAGGCTCCAGCTCCGGCGCCGGCGCCAGCTATGGT
AAGTGCTCTGGCACAGGCTCCGGCCCCTGTTCCAGTCCTCGCTCCAGGACCTCCTCAGGCTGTGGCTCCGCCAGCTCCTA
AGCCGACTCAAGCCGGGGAAGGAACGCTTTCTGAAGCTCTGCTCCAGCTGCAATTTGACGACGAAGACTTGGGGGCCCTT
CTCGGTAATTCTACTGACCCCGCCGTATTCACGGATCTGGCGAGCGTCGATAATTCCGAGTTCCAACAACTCCTGAATCA
AGGCATACCAGTGGCCCCGCATACCACAGAGCCTATGTTGATGGAATACCCAGAGGCCATAACTAGACTGGTAACTGGCG
CGCAACGGCCGCCTGACCCGGCTCCGGCACCTCTGGGGGCACCTGGGTTGCCCAACGGTCTTCTGTCTGGGGACGAAGAC
TTTTCTAGCATTGCGGATATGGATTTCTCAGCTCTGC
(RTA) TGGGCAGCGGCTCCGGCTCTCGTGACTCTCAGAAGGGCATGTTTCTCCCGAAGCCGGAAGCTGGAAGTGC
GATAAGCGACGTTTTTGAAGGCAGAGAGGTGTGTCAACCAAAAAGAATTCGCCCCTTTCACCCACCAGGAAGCCCGTGGGCGA
ACCGCCCGTTGCCGGCctcCCTCGCTCCAACGCCTACTGGACCCGTCCATGAACCCGTTGGCAGTCTCACGCCTGCACCAGTC
CCCCAACCTCTTGATCCAGCCCCCGCTGTCACCCCTGAAGCTTCACATCTTCTTGAAGACCCGGACGAGGAAACGAGCCAAGC
CGTCAAAGCTCTGCGCGAAATGGCAGACACGGTCATACCCCAAAAGGAAGAGGCAGCCATATGCGGACAGATGGATTTGTCCC
ATCCCCCGCCCCGAGGTCACCTTGACGAGCTGACTACTACCCTGGAAAGCATGACGGAAGACCTCAATCTGGACAGTCCCCTT
ACGCCGGAACTGAACGAAATTTTGGACACGTTTCTGAATGATGAATGCCTCCTGCACGCAATGCATATAAGCACGGGGCTGTC
TATCTTTGACACGTCACTCTTTTGA
(terminator)attccgataacttgtttattgcagcttataatggttacaaataaagcaatagcatcacaaatttcacaaat
aaagcatttttttcactgcattctagttgtggtttgtccaaactcatcaatgtatcttatcatgtctg
```

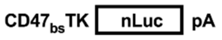

The nanoluciferase coding sequence was amplified from pNL1.1 (Promega) with the following primer in order to include the minimal TK promoter and the CD47 target site:

For primer nLuc	GTGAAAGCAAAGAGGAGAAAAGTAGAGAGAGAGGACAGTGGGG
Rev primer nLuc	TTGGGTATGTCACATTTCATTTCGTATTTTATTTCGCCAGAATGCGTTCGCACA

The oligonucleotides used

V0, original published sequence

5’ GAGGAGAAAAGTAGAGAGAGAGGACAGTTTTTTGACAGGAGAGAGAGATGAAAAGAGGAG

V1, PS(*) binding site (T)$_5$ linker

5’GCTCAAATTACAACGGTATATATC*C*T*G*C*C*A*G*T*C*A*G*TTTTTGAGGAGAAAAGTAGAGAGAGAGGACAGTTTTTTGACAGGAGAGAGAGATGAAAAGAGGAG

V2, PS(*) binding site C18 linker

5’GCTCAAATTACAACGGTATATATC*C*T*G*C*C*A*G*T*C*A*G/iSp18/TTTTTGAGGAGAAAAGTAGAGAGAGAGGACAGTTTTTTGACAGGAGAGAGAGATGAAAAGAGGAG
